# *De Novo* Duplication of 11p15 Associated With Congenital Diaphragmatic Hernia

**DOI:** 10.3389/fped.2018.00116

**Published:** 2018-04-25

**Authors:** Gabriel C. Dworschak, Hartmut Engels, Jessica Becker, Lukas Soellner, Thomas Eggermann, Florian Kipfmueller, Andreas Müller, Heiko Reutter, Martina Kreiß

**Affiliations:** ^1^Institute of Human Genetics, University of Bonn, Bonn, Germany; ^2^Department of Pediatrics, Children's Hospital, University of Bonn, Bonn, Germany; ^3^Institute of Human Genetics, RWTH Aachen, Aachen, Germany; ^4^Department of Neonatology and Pediatric Intensive Care, Children's Hospital, University of Bonn, Bonn, Germany

**Keywords:** congenital diaphragmatic hernia, CDH, Beckwith-Wiedemann syndrome, BWS, 11p15 duplication, partial trisomy, methylation status, *de novo*

## Abstract

**Background:** Congenital diaphragmatic hernia (CDH) is a rare defect of the diaphragm commonly associated with high morbidity and mortality due to lung hypoplasia and pulmonary hypertension. Although in 70% of patients the etiology of a CDH remains unknown, a multitude of causative chromosomal aberrations has been identified.

**Case presentation:** We describe the first case of isolated 11p15 duplication with CDH. The 18.6 Mb large duplication affected 285 RefSeq genes and included the Beckwith–Wiedemann (BWS)-associated imprinting control region 2 (ICR2, *KCNQ1OT1* TSS DMR), whereas the ICR1 (*H19* TSS DMR) was not affected. We were able to demonstrate *de novo* occurrence of the duplication. The paternal origin of the chromosomal material was detected by methylation testing the ICR2. Corresponding to other patients with duplications of the paternal ICR2 copy, a BWS phenotype is not present.

**Conclusions:** The patient presented here together with the review of four other cases from the literature indicate an association between duplications of the chromosomal region 11p15 and developmental defects of the diaphragm. Thus, we suggest duplications of 11p15 as a rare cause of CDH. This association may or may not appear in the context of BWS depending on the extent of the duplication and the imprinting status. Hence, a genetic workup should be performed in patients with CDH, particularly when other abnormalities are noted.

## Introduction

Congenital diaphragmatic hernia (CDH) is a congenital defect of the structural integrity of the diaphragm commonly associated with high morbidity and mortality due to lung hypoplasia and pulmonary hypertension [1]. The defect may range from a small orifice to complete absence of diaphragm leading to protrusion of abdominal contents into the thoracic cavity interfering with normal development of the lungs. Birth prevalence ranges from 1 in 3,000 to 4,000 live births [2]. CDH presents in 50–60% of patients in an isolated way, whereas in the remainder it is associated with additional malformations, commonly affecting the central nervous system, cardiovascular system, genitourinary system, and the gastrointestinal tract [3]. Most cases occur sporadically and genetic as well as environmental factors play a role in the etiology. Evidence for a genetic cause of CDH is provided by animal models [4], the fact that CDH is a feature of several defined genetic syndromes, and its association with chromosomal aberrations [5, 6]. However, in approximately 70% of patients, the etiology of CDH remains unknown [7]. In this report, we present a patient with CDH, mild facial dysmorphism, hepatomegaly, and a *de novo* chromosomal duplication of 11p15 including the imprinting control region 2 (ICR2, KCNQ1OT1 TSS DMR) and compare it to reported patients from the literature.

## Case presentation

**Figure 1:**
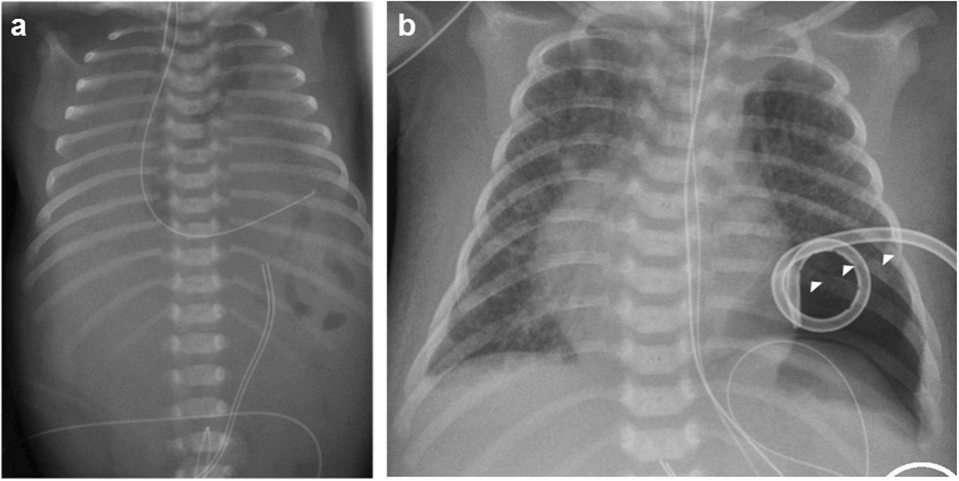
Preoperative **(a)** and postoperative x-rays **(b)** of the index patient. Arrowheads mark the pneumothorax in **(b)**.

**Figure 2:**
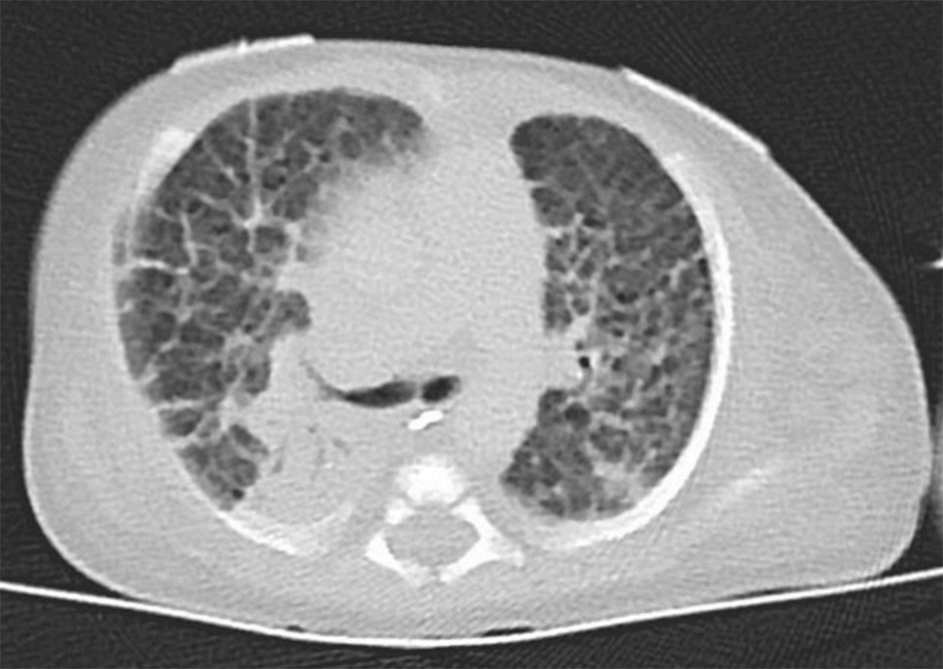
Thoracal CT-scan at 7 weeks showed an interstitial opacity indicating a chronic pulmonary disease.

The index patient was the second child to a 29-year-old mother. The 1-year old sister and the parents were healthy. Further family history was unremarkable and the parents were nonconsanguineous. In the first trimester increased nuchal translucency, polyhydramnios, generalized edema, and left-sided CDH were noted. Prenatally, the diaphragmatic hernia was estimated with o/e LHR 29% (observed to expected lung to head ratio) and liver herniation (“liver up”). The mother gave birth at 34 + 4 weeks of gestation due to premature labor. The girl's birth weight was 2,500 g (60th percentile), length was 44 cm (20th percentile), and head circumference was 32.5 cm (60th percentile). Immediately after delivery, she was intubated and ventilated. Diagnosis of left-sided CDH was confirmed by postnatal chest X-ray (Figure 1a). Upon admission to the neonatal intensive care unit, hemoglobin oxygen saturation was 70% despite FiO_2_ 1.0 and high peak inspiratory pressures on conventional ventilation. Despite aggressive intensive care support including sedation, intensive ventilation with inhaled nitric oxide, infusion of dobutamine, milrinone, and noradrenaline, the patient's respiratory failure continued. In the fourth hour of life, extracorporeal membrane oxygenation (ECMO) was initiated. ECMO stabilized the respiratory condition and eventually could be ceased after an increase of tidal volume over the following 10 days. At the twelfth day of life, CDH repair was successfully performed using a Gore-Tex® patch (Figure 1b). Further dysmorphic features included mild facial dysmorphisms, namely hypertelorism, epicanthus, thin upper lip, and long and narrow feet. Furthermore, the patient had marked hepatomegaly. Yet, she neither showed macroglossia nor macrosomia. Postoperative course was complicated by a valvular pneumothorax requiring drainage for 14 days (Figure 1b). Several bronchoscopic interventions became necessary in relapsing aggravations of respiratory condition. A CT-scan at 7 weeks showed an interstitial opacity indicating a chronic pulmonary disease (Figure 2). Eventually, the patient suffered a sepsis requiring aggressive treatment including catecholamines, intensive ventilation, and antibiotic therapy. Despite all measures, pulmonary and cardiac failure progressed. Because the likelihood of survival was felt to be low, her parents declined further intensive care. Comfort care was initiated, and the patient deceased in her mother's arms at age 9 weeks.

During the pregnancy of the index patient, amniocentesis, and ensuing conventional karyotyping were performed when decompressive puncture became necessary due to polyhydramnios. The result suggested a structurally aberrant chromosome 11. Postnatal karyotyping identified a duplication of 11p15 (46,XX,dup(11)(p15.5p15.1)). To test whether the chromosomal aberration had occurred *de novo*, karyotyping of the parents was performed. Cytogenetic analysis was unremarkable in both parents, indicating a *de novo* event in the patient. Since the resolution of conventional karyotyping was insufficient to assess an involvement of the BWS- and Silver-Russel syndrome-critical region (BWS/SRSCR), we performed oligo-array-CGH (SurePrint G3 ISCA CGH+SNP 180k; Agilent; Santa Clara, CA). Results refined the size of the duplicated region to 18.6 Mb. The duplication affected 285 RefSeq genes, including *KCNQ1* and *CDKN1C* in the ICR2 but not *H19* and *IGF2* in the ICR1 in 11p15 (arr[GRCh37] 11p15.5p15.1[2349955_20939091]x3 dn).

Methylation-sensitive multiplex ligation dependent probe amplification was performed to investigate the parental origin of the duplication (kit ME030-C1, MRC-Holland, Amsterdam, Netherlands). The probes for the *KCNQ1OT1* TSS DMR (ICR2) confirmed the duplication and the methylation-specific probes showed hypomethylation, indicating that the paternal allele was affected by the duplication. The probes for *H19/IGF2* IG-DMR (ICR1) showed a normal copy number and methylation pattern.

## Discussion

**Figure 3:**
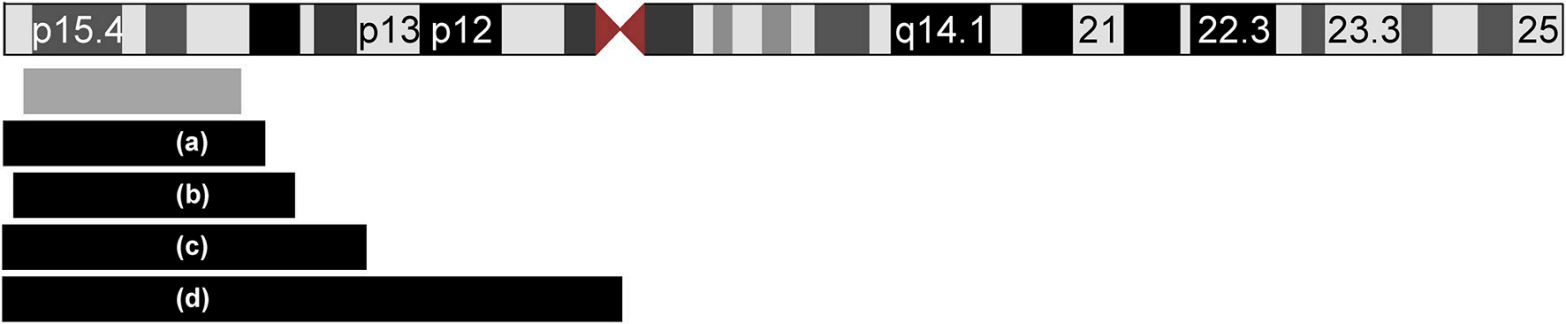
Ideogram of chromosome 11 and extent of the duplications of 11p15 in five patients with CDH. The location and extent of the isolated *de novo* 11p15.1–p15.5 duplication in our patient is depicted with a gray box. Four additional patients from the literature with unbalanced translocations are displayed with black boxes **(a–d)**: **(a)** Case ID 4247 of the ECARUCA database with duplication 11p15-pter (http://ecaruca.radboudumc.nl); **(b)** Chen et al. 11p14.3-p15.5 [13]; **(c)** Turleau et al. 11p14-pter [11]; **(d)** Fryns et al. 11p11-pter [12]. The ideogram is modified from UCSC genome browser.

In this report, we present a patient with CDH, mild facial dysmorphism and hepatomegaly, and a *de novo* duplication of 11p15.5p15.1. To the best of our knowledge, we report here the smallest duplication on the short arm of chromosome 11 which is associated with CDH. The duplication is not combined with another chromosomal imbalance, facilitating a clear genotype-phenotype correlation. Previous studies have implicated chromosomal aberrations in the etiology of CDH [5, 8–10] demonstrating locus heterogeneity. A search of the literature and databases for further cases with CDH and overlapping duplications revealed four reports (Figure 3 and Table 1). In all reported cases, the duplication 11p15 was accompanied by a second chromosomal imbalance.

**Table 1:** Overview of published cases with CDH and duplication 11p15 and coincidence with BWS.

	**CDH**	**Duplication 11p15 (inheritance)**	**BWS**
Our case	+	+ (*de novo*)	–
Turleau et al. [11]	+^b^	+^*^ (pat)	+
Fryns et al. [12]	+	+^*^ (pat)	–
ECARUCA^a^	+	+^*^ (pat)	n.d.
Case ID:4247			
Chen et al. [13]	+	+^*^ (pat)	+

n.d., not specifically discussed by the authors; pat, paternally inherited;

a http://ecaruca.radboudumc.nl;

b congenital diaphragmatic eventration;

* *non-isolated duplications*.

In 1984, Turleau et al. reported a stillborn with an abnormal tongue, posterior diaphragmatic eventration, inner organ congestion with a duplication of 11p15 that was due to a t(4;11)(q33;p14)pat; the authors diagnosed BWS in this fetus based on the duplication and the clinical findings [11]. One year later, Fryns et al. identified a fetus with multiple congenital anomalies including CDH but no feature of BWS and a duplication of 11p11-pter [12]. Karyotyping showed 46,XY,rec(11)dup(11p)inv(11)(p11q25)pat. The third case (case ID: 4247) was reported in the ECARUCA database (http://ecaruca.radboudumc.nl) and presented with CDH and other features including low-set ears, hypertelorism, blepharophimosis, flat and wide nasal bridge, short neck, hypospadias, cryptorchid testes, hydronephrosis, short and bowed femur, and congenital vertical talus. At 29 + 4 weeks of gestation, birth weight was 1,960 g (97 th percentile), body length 42 cm (75 th percentile), and the head circumference 30.3 cm (86 th percentile). The dataset does not indicate if BWS was considered likely. Karyotyping revealed an unbalanced translocation 46,XY,der(2)t(2;11)(p25;p15)pat. Finally, Chen et al. [13] recently reported a male fetus with overgrowth, enlarged placenta and CDH leading to a prenatal diagnosis of BWS. Conventional karyotyping revealed the finding of 46,XY,add(11)(q24.2)dn that was refined by array CGH to a 25 Mb duplication of 11p15.5p14.3 including the BWS/SRSCR and a 5.3 Mb deletion of 11q24.3q25. A paternal origin of the duplication and a *de novo* occurrence was confirmed. However, it cannot be excluded that the phenotypes of the latter four patients (index patients from Turleau et al. [11], Fryns et al. [12], Chen et al. [13], and ECARUCA patient 4247) are at least partially caused by their concurrent imbalances, e.g. the deletion of 4q33-qter, deletion of 11q25-qter, deletion of 2p25-pter and deletion of 11q24.3-q25. Interestingly, we found another report of a fetus with the clinical diagnosis of BWS (macroglossia, omphalocele, visceromegaly, and extra-lobar lung sequestration) and left-sided CDH [14]. Although the authors reported a normal karyotype, a chromosomal aberration below the resolution of conventional karyotyping could have been missed in this case. On the other hand, we found reports of overlapping duplications in patients without CDH: In the public DECIPHER database (http://decipher.sanger.ac.uk/) 10 further patients with overlapping duplications ranging between 1 and 20 Mb and available clinical information are listed. Amongst these, short stature and intellectual disability are recurrent features, but no defect of the diaphragm is noted.

Involvement of one of the two imprinting control regions (ICR1 and ICR2) associated with BWS required further investigation in the presented case: The duplication of the paternal ICR2 allele without obvious BWS features corresponds to the observation that a ICR2 duplication may affect the phenotype only in case the maternal allele is involved [15]. Thus, our case supports this finding and stays in accordance with a recent report on ICR duplications in BWS [16]. Therefore, our extensive genetic workup and the lack of certain clinical features, such as macroglossia, macrosomia, or omphalocele, make the diagnosis of BWS in our case highly unlikely. Although, we did not observe findings suggestive of BWS in our patient, CDH has been discussed in the context of BWS [14]. The finding of patients with 11p15 duplications with CDH is not consistently coinciding with BWS (Table 1). In fact, only two of the four cases from the literature with CDH and 11p duplication presented with BWS [11, 13]. The infrequent occurrence of BWS depends most likely on modifying factors that may include methylation status, extent of duplication, parental origin, and single nucleotide variants that have not been investigated in those reports. However, we have to highlight that the association between 11p15 duplications and CDH are only based on few reports and further investigations are necessary to replicate the findings.

## Concluding remarks

The presented patient and the review of four published cases indicate an association between duplication of 11p15 and developmental defects of the diaphragm. Hence, a genetic workup should be performed in patients with CDH, particularly when another phenotypical abnormality is noted. Furthermore, we describe a co-occurance with BWS caused by 11p duplications and we suggest thorough clinical and genetic workup to differentiate between the 11p duplications within and outside the BWS spectrum.

## Ethics statement

The ethic committee at the Medical Faculty of the University of Bonn has approved the study and parents of the patient gave written informed consent in accordance with the Declaration of Helsinki. The patient's parents gave written informed consent for publication of this report.

## Author contributions

GD wrote the manuscript and performed literature search; HR supervised the entire process of manuscript preparation; AM, FK, and HR were involved in the medical care of the patient; MK, JB, LS, TE, and HE performed the genetic testing. All authors read and approved the final manuscript.

### Conflict of interest statement

The authors declare that the research was conducted in the absence of any commercial or financial relationships that could be construed as a potential conflict of interest. The reviewer NS and handling Editor declared their shared affiliation.

## Abbreviations

BWS: Beckwith-Wiedemann syndrome
CDH: congenital diaphragmatic hernia
ECMO: extracorporeal membrane oxygenation
ICR1: imprinting control region 1
ICR2: imprinting control region 2.
